# Involvement of CRMP2 in Regulation of Mitochondrial Morphology and Motility in Huntington’s Disease

**DOI:** 10.3390/cells10113172

**Published:** 2021-11-15

**Authors:** Tatiana Brustovetsky, Rajesh Khanna, Nickolay Brustovetsky

**Affiliations:** 1 Department of Pharmacology and Toxicology, Indiana University School of Medicine, Indianapolis, IN 46202, USA; tbrousto@iupui.edu; 2 Department of Pharmacology, College of Medicine, University of Arizona, Tucson, AZ 85721, USA; rkhanna@arizona.edu; 3 Center for Innovation in Brain Sciences, University of Arizona, Tucson, AZ 85721, USA; 4 Stark Neurosciences Research Institute, Indiana University School of Medicine, Indianapolis, IN 46202, USA

**Keywords:** Huntington’s disease, neuron, mitochondria, morphology, motility, CRMP2

## Abstract

Mitochondrial morphology and motility (mitochondrial dynamics) play a major role in the proper functioning of distant synapses. In Huntington’s disease (HD), mitochondria become fragmented and less motile, but the mechanisms leading to these changes are not clear. Here, we found that collapsin response mediator protein 2 (CRMP2) interacted with Drp1 and Miro 2, proteins involved in regulating mitochondrial dynamics. CRMP2 interaction with these proteins inversely correlated with CRMP2 phosphorylation. CRMP2 was hyperphosphorylated in postmortem brain tissues of HD patients, in human neurons derived from induced pluripotent stem cells from HD patients, and in cultured striatal neurons from HD mouse model YAC128. At the same time, CRMP2 interaction with Drp1 and Miro 2 was diminished in HD neurons. The CRMP2 hyperphosphorylation and dissociation from Drp1 and Miro 2 correlated with increased fission and suppressed motility. (S)-lacosamide ((S)-LCM), a small molecule that binds to CRMP2, decreased its phosphorylation at Thr 509/514 and Ser 522 and rescued CRMP2’s interaction with Drp1 and Miro 2. This was accompanied by reduced mitochondrial fission and enhanced mitochondrial motility. Additionally, (S)-LCM exerted a neuroprotective effect in YAC128 cultured neurons. Thus, our data suggest that CRMP2 may regulate mitochondrial dynamics in a phosphorylation-dependent manner and modulate neuronal survival in HD.

## 1. Introduction

Huntington’s disease (HD) is a hereditary neurodegenerative disorder linked to a single mutation in exon 1 of the *Htt* gene, encoding protein huntingtin (HTT) [1]. This mutation results in an increased number of CAG repeats (>35) in the *Htt* gene and subsequently to the elongation of polyglutamine stretch in HTT [2]. How exactly this mutation leads to HD pathology is not clear, but in early studies, mitochondrial dysfunction was proposed to be a contributing factor in HD pathogenesis [3,4,5,6,7,8,9,10]. However, in recent years, the role of mitochondrial dysfunction in HD pathogenesis was questioned, and compelling data indicating the lack of overt bioenergetic deficits in brain mitochondria in pre-symptomatic and early symptomatic mouse models were provided [11,12,13,14,15,16,17,18,19]. This strongly suggested that mitochondrial bioenergetic dysfunction most likely does not contribute to HD pathogenesis and could be secondary to other pathogenic mechanisms.

On the other hand, alterations in mitochondrial morphology and motility were reported in different HD mouse models and were proposed to play an important role in HD pathology [18,20,21,22,23]. In HD, neuronal mitochondria become shorter, suggesting a shift between fission and fusion toward increased mitochondrial fragmentation [20,21,24,25]. In addition, mitochondrial traffic is stalled in HD neurons [18,20,22,23,26]. Despite tremendous interest in these alterations, the exact mechanisms underlying the HD-associated changes in mitochondrial dynamics are not completely understood. The mechanisms involved in regulating mitochondrial morphology and motility include different proteins with various functions. For example, large GTPase Drp-1 is involved in mitochondrial fission [27,28], whereas the proteins Mitofusin 1 and 2 are involved in mitochondrial fusion [29]. Kinesins and dyneins are involved in mitochondrial motility and can interact with CRMP2 [30,31], whereas adaptor proteins such as Miro and Milton connect mitochondria to kinesins and dyneins [32,33].

Collapsin response mediator proteins (CRMPs) belong to a family of cytosolic phosphoproteins (CRMP1–5) that are predominantly expressed in the developing brain [34]. CRMPs are involved in modulating microtubule polymerization, actin bundling, and endocytosis, resulting in neuronal differentiation. CRMP2 is a cytosolic phosphoprotein implicated in axon guidance and neurite outgrowth via the Semaphorin3A pathway [35,36,37]. In contrast to other members of the CRMP family, CRMP2 is maintained at a high level of expression in adults [38]. CRMP2 does not have enzymatic activity, and its actions are mediated by CRMP2 interaction with other proteins [37,39,40], including kinesin-1 and dynein—molecular motors involved in mitochondrial motility [30,31].

CRMP2 interaction with other proteins is regulated by phosphorylation [36,37,40]. Non-phosphorylated CRMP2 is prone to interaction with its binding partners and promotes axon/neurite outgrowth, whereas phosphorylated CRMP2 loses its binding ability and functional activity [41]. CRMP2 can be phosphorylated at different sites by different kinases. Under normal conditions, CRMP2 is phosphorylated up to 30% of the maximal phosphorylation [42]. Rho kinase phosphorylates CRMP2 at Thr 555 [43,44], whereas the src family kinase Fyn phosphorylates CRMP2 at Tyr 32 [45]. Cyclin dependent kinase 5 (Cdk-5) phosphorylates CRMP2 at Ser 522 and readies CRMP2 for subsequent phosphorylation by GSK-3β at Thr 509 and Thr 514 [41,46,47,48]. Changes in CRMP2 phosphorylation can be observed in various neuropathologies. For example, Mokhtar et al. found that CRMP2 is hyperphosphorylated in Alzheimer’s disease (AD) and HD [49].

In the present study, we assessed CRMP2 phosphorylation in postmortem brain tissues from HD patients and unaffected individuals, in cultured human striatal neurons derived from induced pluripotent stem cells (iPSCs) from HD patients and unaffected individuals, as well as in cultured striatal neurons from the transgenic HD mouse model YAC128 and their wild-type littermates. In addition, we investigated the link between the CRMP2 phosphorylation state and mitochondrial morphology and motility in striatal neurons from YAC128 mice. We found that CRMP2 is hyperphosphorylated in HD. This correlated with a decreased interaction of CRMP2 with Drp1 and Miro 2 and with an increased mitochondrial fragmentation and suppressed mitochondrial motility. Preventing CRMP2 hyperphosphorylation with a small-molecule (S)-lacosamide ((S)-LCM) [50,51] restored CRMP2’s interaction with other proteins, rescued mitochondrial morphology and motility, and protected HD mouse neurons from cell death.

## 2. Materials and Methods

### 2.1. Human Postmortem Brain Tissues

Human postmortem striatal brain tissues were obtained from the University of Maryland Brain and Tissue Bank (UMDBTB, Baltimore, MD, USA), which is a Brain and Tissue Repository of the NIH NeuroBioBank. Deidentified clinical information about postmortem brain samples is reported in Supplemental Table S1. The length of CAG repeats in the *Htt* gene of HD patients was determined by Laragen Inc. (Culver City, CA, USA). The samples were stored at −86 °C. Prior to the experiments, samples were placed in an ice-cold solution containing 50 mM Tris-HCl, pH 7.4, 150 mM NaCl, 1 mM EDTA, and supplemented with Phosphatase and Protease Inhibitors Cocktail (Roche, Cat# 04906845001 and Cat# 04693124001) and slowly thawed on ice.

### 2.2. Animals

All procedures with animals were performed in compliance with the US National Institutes of Health Guide for the Care and Use of Laboratory Animals, as well as in accordance with the Indiana University School of Medicine Institutional Animal Care and Use Committee approved protocol (# 11385 MD/R). Transgenic YAC128 mice and wild-type FVB/NJ mice of both sexes were purchased from Jackson Laboratories (Bar Harbor, ME, USA), and breeding colonies were established in the Laboratory Animal Resource Center at Indiana University School of Medicine, Indianapolis, IN. YAC128 mice express full-length human mutant huntingtin (mHTT) containing 128 glutamines in the polyglutamine (polyQ) stretch and wild-type mouse huntingtin (Htt) [52,53]. Male YAC128 mice were bred with female FVB/NJ mice (background strain). The mice were housed under standard conditions with free access to water and food. All mice were housed in polycarbonate cages, with 3 mice per cage.

### 2.3. Genotyping

All offspring were genotyped with a PCR assay on tail DNA. Briefly, PCR of tail DNA was performed in accordance with the protocol provided by Jackson Laboratory using oligonucleotide primers oIMR6533 (GGCTGA GGAAGCTGAGGAG) and TmoIMR1594 (CCGCTCAGGTTCTGCTTTTA) purchased from Invitrogen (Carlsbad, CA, USA). The PCR reaction mixture contained 1 µL of DNA template and 23 µL of Platinum PCR SuperMix (Invitrogen) supplemented with 0.39 µM of each primer (Invitrogen); the total volume was 25 µL. Cycling conditions were 5 min at 95 °C, 35 cycles at 30 s at 95 °C, 30 s at 56 °C, 60 s at 72 °C, and 10 min at 72 °C. Reaction products were analyzed on 1.2% agarose gel run at 100 V for 60 min with Tris-acetate-EDTA running buffer containing 1X GelRed^TM^ Nucleic Acid Gel Stain (Biotium, Fremont, CA, USA).

### 2.4. Mouse Cell Culture

Primary culture of mouse striatal neurons was prepared from postnatal day 1 FVB/NJ mouse pups in accordance with the IACUC-approved protocol and procedures published previously [54]. We used neuronal-glial co-cultures derived from postnatal day 1 mouse pups as they are more physiologically relevant and approximate more mature, better developed cells than pure neuronal culture derived from embryonic animals. For fluorescence recordings, neurons were plated on glass-bottomed Petri dishes as previously described [54]. For all platings, 35 μg/mL of uridine plus 15 μg/mL of 5-fluoro-2′-deoxyuridine were added 24 h after plating to inhibit the proliferation of microglia. Cultures were maintained in a 5% CO_2_ atmosphere at 37 °C in MEM supplemented with 10% NuSerum (BD Bioscience, Bedford, MA, USA), 27 mM glucose. Mouse cell cultures were used at 10–12 days in vitro (10–12 DIV). For cell death assessment, cell cultures were used at 21 DIV.

### 2.5. Generation of Human Striatal Neurons from Induced Pluripotent Stem Cells (iPSCs) from HD Patients and Unaffected Individuals

The generation of iPSC-derived neural progenitors and neural differentiation from neural progenitors was performed as we described previously [16]. In our study, we used undifferentiated human iPSCs derived from fibroblasts of control, unaffected individuals (control) and HD patients. The size of CAG repeats was determined by Laragen (Culver City, CA, USA), a company used previously by us [16] and other investigators for CAG repeat sizing [55]. The undifferentiated human control iPSCs were provided by Dr. George Daley (Harvard University, Cambridge, MA, USA; hFib2-iPS5 cell line, 21/18 CAG repeats according to Laragen), by Dr. David Gamm (University of Wisconsin, Madison, WI, USA; TiPS5 cell line, 29/22 CAG repeats according to Laragen), and by Dr. Jason Meyer (IUPUI, Indianapolis, IN; miPS-2 cell line, 21/19 CAG repeats according to Laragen). The undifferentiated human HD iPSCs were provided by Dr. George Daley (Harvard University, Cambridge, MA, USA; HD-iPS4 cell line, 74/19 CAG repeats according to Laragen) and by Cedars-Sinai Medical Center, Los Angeles, CA (CS04iHD-46n10, 49/28 CAG repeats, and CS03iHD-53n2, 59/18 CAG repeats according to Laragen, cell lines). The findings presented in our manuscript were confirmed using control human neurons and HD human neurons produced from three control undifferentiated human iPSCs lines and three undifferentiated human HD iPSCs lines.

### 2.6. Immunoblotting

Samples of postmortem brain tissues (striatum) from HD patients and unaffected individuals were homogenized in a solution containing 50 mM Tris-HCl, pH 7.4, 150 mM NaCl, 1% NP-40, 0.1% SDS, 1 mM EDTA, and supplemented with Phosphatase and Protease Inhibitors Cocktail (Roche, Indianapolis, IN, USA, Cat# 04906845001 and Cat# 04693124001). The homogenates were incubated for 30 min on ice and then centrifuged at 100,000× *g* 30 min. The pellet was discarded, and the supernatant was used for gel electrophoresis. Human neurons derived from iPSCs from HD patients and unaffected individuals, cultured on 35 mm Petri dishes for 60 days after conversion [16], and mouse striatal neurons cultured on 35 mm Petri dishes for 12 DIV were prepared for gel electrophoresis in a similar way to samples of postmortem brain tissues. Bis-Tris gels (4–12%, Invitrogen, Carlsbad, CA, USA, Cat# NP0335) were used to separate proteins via electrophoresis (20 µg protein/lane). Following electrophoresis, proteins were transferred to a Hybond-ECL nitrocellulose membrane (Amersham Biosciences, Piscataway, NJ, USA, Cat# RPN78D). Blots were incubated at room temperature for 1 h in a blocking solution composed of either 5% BSA phosphate-buffered saline, pH 7.2, plus 0.15% Triton X-100, or 5% milk, phosphate-buffered saline, pH 7.2, plus 0.15% Triton X-100 for total protein immunoblotting. For phosphoprotein immunoblotting, 5% BSA, Tris-HCl buffered saline, pH 7.2, plus 0.15% Triton X-100 was used. After blocking, blots were incubated with either rabbit anti-CRMP2 (Sigma, St. Louis, MO, USA, Cat # C2993, 1:1000), sheep anti-CRMP2 pThr 509/514 (KinaSource, Dundee, UK, Cat # PB-043, 1:1500), rabbit anti-CRMP2 pSer522 (ECM Biosciences, Cat # CP2191, 1:1500), mouse anti-GAPDH (Abcam, Cambridge, MA, USA, Cat # ab9484, 1:2000), rabbit anti-Drp1 pSer 616 (Cell Signaling, Danvers, MA, USA, Cat# 3455, 1:1000), mouse anti-β-actin (ThermoFisher Scientific, Carlsbad, CA, USA, Cat# MA5-15739, 1:1000), or rabbit anti-Miro 2 (Proteintech, Rosemont, IL, USA, Cat # 11237-1-AP, 1:1000) antibodies. Blots were subsequently incubated with either goat anti-mouse or goat anti-rabbit IgG (1:25,000 or 1:20,000, respectively) coupled with horseradish peroxidase (Jackson ImmunoResearch Laboratories, West Grove, PA, USA) and developed with Supersignal West Pico chemiluminescent reagents (Pierce, Rockford, IL, USA, Cat# 32106). Molecular mass marker Page Ruler Plus Prestained Protein Ladder (5 μL, ThermoFisher Scientific; Cat# 26619) was used for the molecular mass determination of the bands. The immunoblot images were inverted, and the integrated density of bands was measured after background subtraction using Adobe Photoshop 22.2.0. (Adobe Inc., San Jose, CA, USA).

### 2.7. Co-Immunoprecipitation

Mouse striatal neurons in culture (10–12 DIV) were incubated with 10 μM (S)-lacosamide (the functionalized amino acid (S)-2-acetamido-N-benzyl-3-methoxypropionamide ((S)-lacosamide, (S)-LCM)) was purchased from Sigma) for 7 days. Then, cells were lysed in the lysis buffer, containing 139 mM NaCl, 20 mM Tris-HCl, Proteinase Inhibitor Cocktail (Roche), 1% NP40, and 0.1% SDS. Co-immunoprecipitation experiments were performed on freshly prepared cell lysates from mouse striatal neuronal cultures at 12–14 DIV. Lysates were clarified to remove any additional precipitate by incubating with Protein A/G agarose beads (Santa Cruz Biotechnology, Paso Robles, CA, USA, Cat# sc-2002, Santa Cruz, CA, USA) for 2 h at 4 °C. The lysates were then incubated overnight with primary rabbit anti-CRMP2 (Sigma, Cat # C2993, 1:1000), rabbit anti-Miro 2 (Proteintech, Cat # 11237-1-AP, 1:500), rabbit anti-DRP1 (Santa Cruz Biotechnology, Cat# sc-32898, 1:100), rabbit anti-FIS1 (ThermoFisher Scientific, Cat# 10956-1-AP, 1:100), mouse anti-MFF (Santa Cruz Biotechnology, Cat# sc-398731, 1:500), rabbit anti-syntaphilin (ThermoFisher Scientific, Cat# 13646-1-AP, 1:500), or rabbit anti-syntabulin (ThermoFisher Scientific, Cat# 16972-1-AP, 1:500) antibodies under gentle agitation at 4 °C followed by incubation with Protein A/G agarose beads (Santa Cruz Biotechnology, Cat# sc-2002) for 2 h at 4 °C. The immune-captured complexes were then washed three times with lysis buffer before being heated at 70 °C in equal volumes of SDS loading dye (Invitrogen, Carlsbad, CA, USA). In co-immunoprecipitation experiments, Tris-Acetate gels (3–8%, Invitrogen, Cat# EA0375BOX) were used to separate proteins via electrophoresis (20 µg protein/lane). Samples were then processed by immunoblotting as previously described [56,57]. Blots were probed with rabbit anti-CRMP2, rabbit anti-Drp1, rabbit anti-Miro 2, anti-FIS1 (mitochondrial fission 1 protein), rabbit anti-MFF (Mitochondrial fission factor), rabbit anti-syntaphilin, or rabbit anti-syntabulin antibodies (each diluted 1:1000). All blots represent at least 3 separate, independent experiments. The immunoblot images were inverted, and the integrated density of bands was measured after background subtraction using Adobe Photoshop 22.2.0.

### 2.8. Neuronal Transfection

To visualize mitochondria within live cells, cultured striatal neurons were transfected in suspension during plating using electroporator BTX 630 ECM (Harvard Apparatus, Holliston, MA, USA) with a plasmid encoding mitochondrially targeted enhanced yellow fluorescent protein (mito-eYFP, generously provided by Dr. Roger Tsien, UCSD). The electroporation was performed in the electroporation cuvettes with a 2 mm gap in a low voltage mode: voltage 100 V, capacitance 475 μF, resistance 25 Ω. In total, 8 μg of DNA was used per a single electroporation. The electroporation procedure usually provided an approximate transfection rate of 10%in primary cultures of mouse striatal neurons [58]. The transfected neurons were imaged 10–12 days after transfection.

### 2.9. Mitochondrial Morphology

Mitochondrial morphology in live cultured striatal neurons was analyzed at room temperature (23 °C) as described previously [59]. Briefly, serial images of neuronal mitochondria visualized with mito-eYFP were collected using spinning-disk confocal microscopy. For this purpose, a Nikon Eclipse TE2000-U inverted microscope equipped with a Yokogawa spinning-disk confocal unit CSU-10, a back-thinned EM-CCD camera Andor iXon^EM^+ DU-897 (Andor Technology, South Windsor, CT, USA), and a motorized flat-top stage Prior H-117 (Prior Scientific, Rockland, MA, USA) was used. This setup was controlled by Andor iQ 1.4 software (Andor Technology, South Windsor, CT, USA). To visualize mitochondria, neurons were illuminated at 488 nm using an air-cooled Kr/Ar laser T643-RYB-A02 (Melles Griot, Carlsbad, CA, USA). The laser power was set to the minimum level (<5%) which was sufficient to provide high-quality images while preventing excessive photobleaching. Fluorescence was collected through a 505 nm dichroic mirror and a 535 ± 25 nm emission filter using an objective Nikon CFI Plan Apo 100 × 1.4 NA. Serial images (z-stacks) were collected using the piezoelectric positioning device PIFOC^®^ P-721 (Physik Instrumente, Auburn, MA, USA) with a z-step of 0.1 μm. While imaging the whole mitochondrial network within neuronal somata, the spatial resolution of the Andor iXon^EM^ + DU-897 camera (pixel size 16 × 16 μM) was increased by installing a 2× extender lens in front of the camera. The 3D blind deconvolution of z-stacks and 3D rendering was performed using AutoDeblur Gold CF 1.4.1 software (MediaCybernetics, Silver Spring, MD, USA). Three-dimensional maximal projection of the mitochondrial network was performed using Imaris 5.7.0 (Bitplane Inc., Saint Paul, MN, USA) as we described previously [59]. To calibrate the image processing and mitochondrial measurements, fluorescent microbeads were used [59]. The length of mitochondria was measured with individual mitochondria located in neuronal processes. Per each experimental condition, 100 randomly chosen mitochondria from at least 10 neurons from 3 different platings were analyzed. During fluorescence measurements, neurons were incubated in the standard bath solution containing 139 mM NaCl, 3 mM KCl, 0.8 mM MgCl_2_, 1.8 mM CaCl_2_, 10 mM NaHEPES, pH 7.4, 5 mM glucose, and 65 mM sucrose. Sucrose was used to maintain an osmolarity similar to that in the growth medium (340 mosm). The osmolarity of the solutions was measured with the osmometer Osmette II™ (Precision Systems Inc., Natick, MA, USA).

### 2.10. Mitochondrial Motility

Mitochondrial motility in striatal cultured neurons was assessed at 37 °C using wide-field fluorescence microscopy. Mitochondrial traffic was recorded with a Nikon Eclipse TE2000-U inverted microscope using a Nikon objective Nikon CFI Plan Apo 100 × 1.4 NA and Photometrics Cool SNAP_HQ_ camera (Roper Scientific, Tucson, AZ, USA) controlled by MetaMorph software 6.3 (Molecular Devices, Downingtown, PA, USA). The excitation light (480 ± 20 nm) was delivered by a Lambda-LS system (Sutter Instruments, Novato, CA, USA), and fluorescence was measured through a 505 nm dichroic mirror at 535 ± 25 nm. The images were acquired during the time-course of the experiment (5 min) with a frequency of 1 Hz. The motility of neuronal mitochondria was analyzed after constructing kymographs using NIH ImageJ 1.53a software.

### 2.11. Cell Death

After culturing mouse striatal neurons for 21 DIV, spontaneous cell death was assessed by Chromatin Condensation/Membrane Permeability/Dead Cell Apoptosis Kit (ThermoFisher Scientific, Cat #V23201), containing Hoechst 33342, YO-PRO-1, and propidium iodide (PI) [60]. Hoechst 33342 staining indicated live cells. Nuclei staining with PI is associated with the loss of barrier properties of the plasma membrane and is considered an indication of necrosis [61]. An induction of apoptosis was evaluated with YO-PRO-1 staining. Dying neurons were detected using a Nikon Eclipse TE2000-U inverted microscope equipped with a Nikon CFI SuperFluor 20 × 0.75 NA objective and a Photometrics cooled CCD camera CoolSNAP_HQ_ (Roper Scientific, Tucson, AZ, USA) controlled by MetaMorph 6.3 software (Molecular Devices, Downingtown, PA, USA). These experiments were performed on neurons from at least three separate platings.

### 2.12. Statistics

Data are displayed as the mean ± SD of the indicated number of separate experiments. Statistical analysis of the experimental results consisted of an unpaired *t-*test or one-way analysis of variance (ANOVA) followed by the Bonferroni post hoc test (GraphPad Prism^®^ version 4.0, GraphPad Software Inc., La Jolla, CA, USA). Every experiment was performed using several different preparations of cultured neurons.

## 3. Results

### 3.1. CRMP2 Hyperphosphorylation in HD

**Figure 1:**
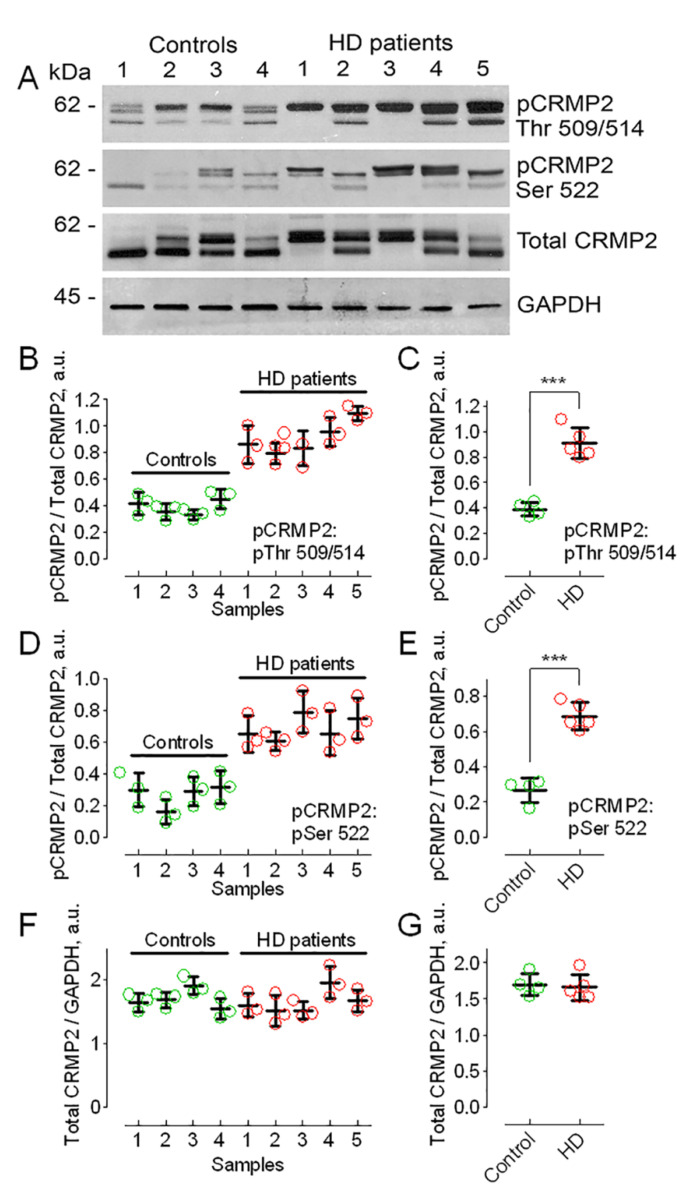
CRMP2 hyperphosphorylation in postmortem brain tissues of HD patients compared to postmortem brain samples of unaffected individuals. (**A**), Western blots with pCRMP2 antibodies for Thr 509/514 and Ser 522, total CRMP2, and GAPDH (loading control). Molecular weight markers (kilodalton, kDa) are indicated in the left margin of the blots. (**B**–**G**), densitometry data. All samples were immunoblotted three times (**B**,**D**,**F**), and then individual data were averaged across the different blots (**C**,**E**,**G**). Here, and in other figures, the data from individual experiments are shown as the colored circles. In (**B**,**D**,**F**), pCRMP2 are normalized by total CRMP2 and total CRMP2 are normalized by GAPDH in 9 individual samples from 4 control and 5 HD human brain samples, respectively. Here, and in other figures, the phosphorylation sites are indicated in the panels. Data are mean ± SD, N = 3 technical replicates (3 separate immunoblotting experiments). In (**C**,**E**), averaged pCRMP2 are normalized by total CRMP2 from individual samples pooled together for statistical analysis. Data are mean ± SD. N = 4–5, data are pooled together from separate immunoblotting experiments with 4 control and 5 HD human brain samples shown in (**B**,**D**), *** *p* < 0.001 comparing brain tissue from HD patients (HD) and unaffected individuals (control). In **G**, total CRMP2 normalized by GAPDH from experiments shown in (**F**). Data are mean ± SD. N = 4–5 separate immunoblotting experiments with 4 control and 5 HD human brain samples.

Previously, it was shown that the hyperphosphorylation of CRMP2 may restrict CRMP2’s interaction with its binding partners and affect CRMP2 functional effects [62]. In our experiments, we assessed total CRMP2 expression and CRMP2 phosphorylation in postmortem brain tissues (putamen) of HD patients and compared these with CRMP2 expression and phosphorylation in postmortem brain tissues from unaffected individuals. Total CRMP2 was unchanged (Figure 1A,F,G), but CRMP2 phosphorylation at Thr 509/514 and Ser 522 in HD brain tissues was higher than in samples from unaffected individuals (Figure 1A–E).

**Figure 2:**
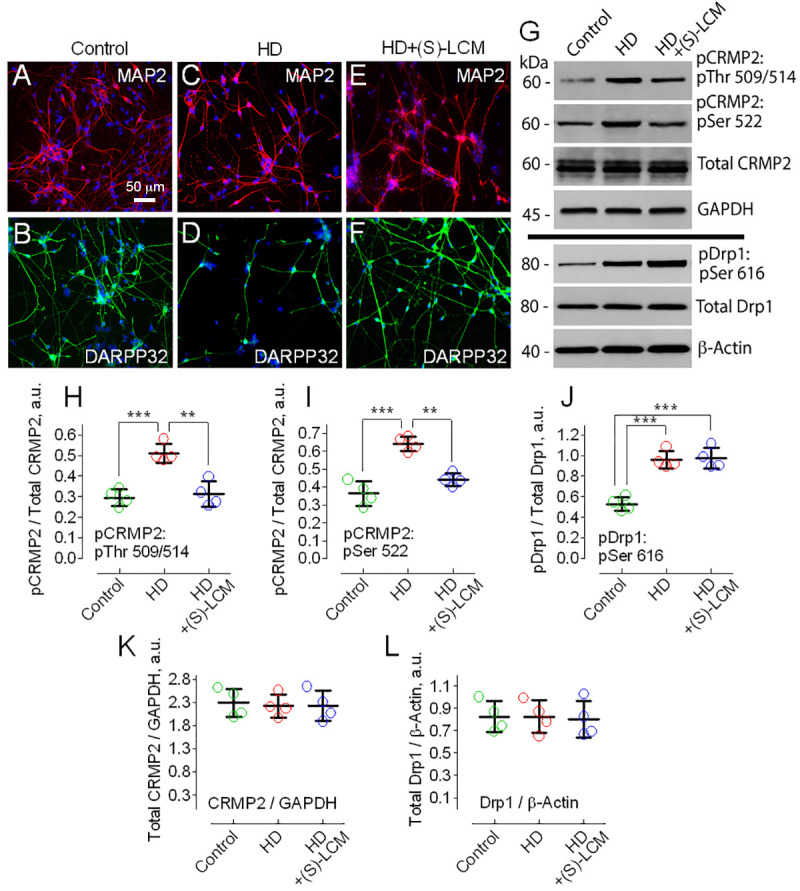
Increased CRMP2 and Drp1 phosphorylation in human striatal neurons derived from induced pluripotent stem cells (iPSCs) from fibroblasts of HD patients compared to striatal neurons from unaffected individuals (control). (S)-lacosamide ((S)-LCM) prevented CRMP2 hyperphosphorylation but failed to diminish Drp1 phosphorylation in HD neurons. In these experiments, human neurons from three control cell lines (hFib2-iPS5, TiPS5, and miPS-2) and three HD cell lines (HD-iPS4, CS04iHD-46n10, and CS03iHD-53n2) were used. With neurons from miPS-2 cell line (control) and CS03iHD-53n2 cell line (HD), immunoblotting experiments were performed twice. Scale bar = 50 µm (**A**,**C**,**E**), representative immunocytochemistry images of MAP2 expression in control, HD, and HD neurons, treated with 10 μM of (S)-LCM for the last 7 days prior to imaging, respectively. (**B**,**D**,**F**), representative immunocytochemistry images of DARPP32 (the striatal marker) expression in control, HD, and HD neurons, treated with 10 μM of (S)-LCM for the last 7 days prior to imaging, respectively. (**G**), representative Western blots of lysates prepared with human neurons from HD patients and unaffected individuals. Where indicated, neurons were treated with 10 µM of (S)-LCM for the last 7 days prior to analysis. GAPDH and β-actin are loading controls. (**H**–**L**), densitometry data. Here and in other Figure legends, the colored circles indicate data from individual measurements. (**H**,**I**), pCRMP2 normalized by total CRMP2. Data are mean ± SD, N = 4, ** *p* < 0.01 comparing HD neurons with and without treatment with (S)-LCM, *** *p* < 0.001 comparing control and HD neurons. (**J**), pDrp1 normalized by total Drp1. Data are mean ± SD, N = 4, *** *p* < 0.001 comparing control with HD and HD + (S)-LCM neurons. (**K**,**L**), total CRMP2 normalized by GAPDH and total Drp1 normalized by β-Actin, respectively, N = 4.

Next, we assessed CRMP2 phosphorylation in human striatal neurons derived from induced pluripotent stem cells (iPSCs) produced from fibroblasts of HD patients and unaffected individuals (Figure 2). We previously reported a detailed characterization of human striatal neurons derived from iPSCs [16]. These neurons expressed microtubule-associated protein 2 (MAP2), a neuronal marker (Figure 2A,C,E), and dopamine- and cAMP-regulated phosphoprotein 32 kDa (DARPP32), a striatal marker (Figure 2B,D,F). Judging from the electrophysiological assessment performed previously [16], the neurons were functionally active. Consistent with the results obtained from postmortem brain tissues, total CRMP2 expression was unchanged (Figure 2G,K), whereas CRMP2 was hyperphosphorylated at Thr 509/514 and Ser 522 in human striatal neurons from HD patients compared to neurons from unaffected individuals (Figure 2G,H,I). In addition to CRMP2, Drp1, a large GTPase involved in mitochondrial fission [27], was hyperphosphorylated at Ser 616 in neurons from HD patients (Figure 2G,J) and this was most likely due to phosphorylation by Cdk-5 and GSK-3β [63,64,65].

**Figure 3:**
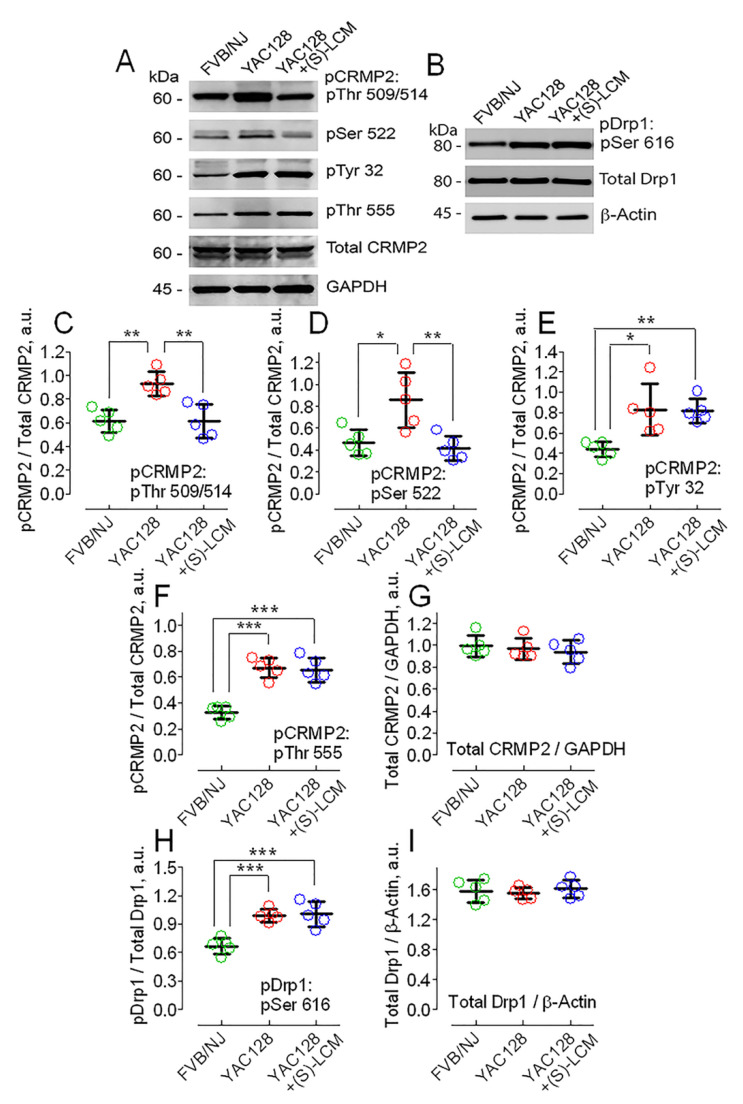
Increased CRMP2 (**A**) and Drp1 (**B**) phosphorylation in cultured striatal neurons from YAC128 mice compared to striatal neurons from FVB/NJ mice. (S)-lacosamide ((S)-LCM) prevented CRMP2 hyperphosphorylation but failed to diminish Drp1 phosphorylation in YAC128 neurons. YAC128 neurons were treated with 10 µM of (S)-LCM for the last 7 days prior to analysis. GAPDH and β-actin are loading controls. In (**A**) and (**B**), representative Western blots are shown. In (**C**–**I**), densitometry data are shown. (**C**–**G**), pCRMP2 normalized by total CRMP2 (**C**–**F**) and total CRMP2 normalized by GAPDH (**G**), respectively. In (**H**,**I**), pDrp1 normalized by total Drp1 and total Drp1 normalized by β-Actin, respectively. Data are mean ± SD, N = 5 separate experiments. (**C**), ** *p* < 0.01, comparing FVB/NJ neurons and YAC128 neurons; comparing YAC128 neurons with and without (S)-LCM treatment. (**D**), * *p* < 0.05 comparing FVB/NJ and YAC128 neurons, ** *p* < 0.01, comparing YAC128 neurons with and without (S)-LCM treatment. (**E**), * *p* < 0.05 comparing FVB/NJ and YAC128 neurons, ** *p* < 0.01, comparing FVB/NJ neurons with YAC128 neurons treated with (S)-LCM. (**F**,**H**), *** *p* < 0.01, comparing FVB/NJ neurons and YAC128 neurons with and without (S)-LCM treatment.

In the following experiments, we extended our study to cultured striatal neurons from YAC128 mice, an established mouse model of HD [66], and from their genetic background FVB/NJ mice. Striatal neurons are the most vulnerable cell type in HD [67,68], and therefore, we were focused on this type of cell. We found increased CRMP2 phosphorylation in cultured striatal neurons from YAC128 mice compared with striatal neurons from FVB/NJ mice (Figure 3). In neurons from YAC128 mice, CRMP2 phosphorylation at Thr 509/514 and Ser 522 was increased, whereas the expression of total CRMP2 remained unchanged (Figure 3A,C,D,G). Thus, CRMP2 expression and the phosphorylation pattern of CRMP2 at Thr 509/514 and Ser 522 in neurons from YAC128 mice was similar to the expression and phosphorylation profile of CRMP2 in postmortem brain tissues from HD patients (Figure 1) and in human striatal neurons derived from iPSCs of HD patients (Figure 2). Further, in neurons from YAC128 mice, CRMP2 phosphorylation at Tyr 32 and Thr 555 was also increased compared with neurons from FVB/NJ mice (Figure 3A,E,F). Since increased Drp1 phosphorylation at Ser 616 could lead to augmented Drp1 recruitment to mitochondria, its overactivation, and increased mitochondrial fission [69], we also assessed Drp1 phosphorylation at Ser 616. We found that the phosphorylation of Drp1 at Ser 616 was increased in neurons from YAC128 mice (Figure 3B,H), consistent with previously reported findings from the HD striatal cell model HdhQ111 compared with control HdhQ7 cells [70]. The expression of total Drp1 was unchanged in neurons from YAC128 mice compared with neurons from FVB/NJ mice (Figure 3B,I).

Previous work demonstrated that the small-molecule (S)-LCM was shown to bind to CRMP2 [50] and block its phosphorylation by Cdk-5 at Ser 522 [51]. We are not aware of other compounds with a similar mechanism of action. Since the phosphorylation of Ser 522 by Cdk-5 primes CRMP2 phosphorylation at GSK-3β sites [46,47], inhibiting the Cdk-5-mediated phosphorylation of CRMP2 may also suppress subsequent CRMP2 phosphorylation by GSK-3β kinase at Thr 509/514. Indeed, in our experiments, (S)-LCM (10 μM for the last 7 days prior to analysis) decreased phosphorylation at Ser 522 and Thr 509/514 in cultured striatal neurons from YAC128 mice compared to neurons from FVB/NJ mice (Figure 3A,C,D), but did not influence phosphorylation at Tyr 32 and Thr 555 (Figure 3A,E,F). In addition, (S)-LCM did not decrease the phosphorylation of Drp1 at Ser 616 (Figure 3B,H). Furthermore, (S)-LCM did not affect the total CRMP2 and total Drp1 expression in neurons from YAC128 mice (Figure 3A,G,I). Similarly, (S)-LCM decreased Ser 522 and Thr 509/514 phosphorylation in human neurons from HD patients (Figure 2G,H,I) but did not affect the total CRMP2 and total Drp1 expression as well as Drp1 phosphorylation at Ser 616 (Figure 2G,J,K,L). In control human or mouse neurons, (S)-LCM did not produce detectable effects (not shown).

### 3.2. CRMP2 Interaction with Proteins Regulating Mitochondrial Dynamics

**Figure 4:**
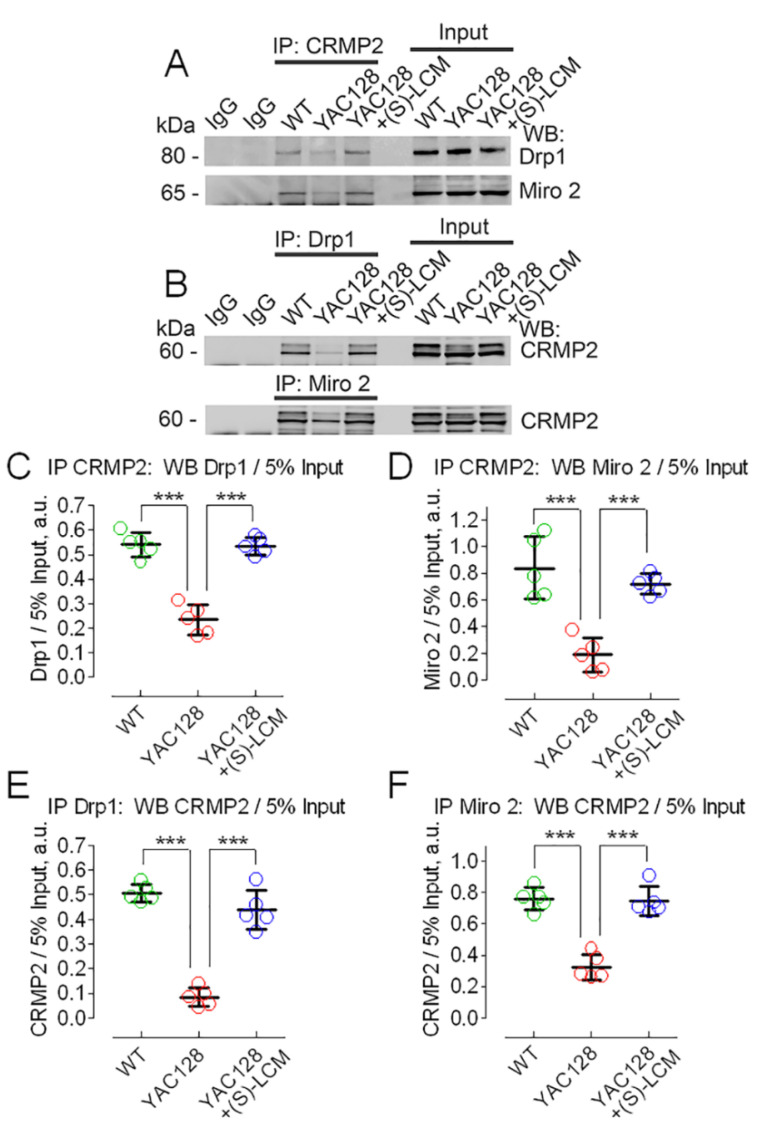
Hyperphosphorylation of CRMP2 in neurons from YAC128 mice correlated with decreased CRMP2 binding to Drp1 and Miro 2, whereas (S)-lacosamide ((S)-LCM) prevented disruption of CRMP2 binding to these proteins. (**A**), immunoprecipitation (IP) with anti-CRMP2 antibody followed by Western blotting (WB) with anti-Drp1 and anti-Miro 2 antibodies; (**B**), IP with anti-Drp1 and anti-Miro 2 antibodies followed by WB with anti-CRMP2 antibody. Where it is indicated, YAC128 neurons were treated with 10 µM of (S)-LCM for the last 7 days prior to analysis. The input was 5% of the total protein used in the pull-down procedure. (**C**–**F**), densitometry data. Drp1, Miro 2, and CRMP2 were normalized by the corresponding 5% input value. Data are mean ± SD. *** *p* < 0.001 comparing FVB/NJ neurons and YAC128 neurons; comparing YAC128 neurons with and without (S)-LCM treatment, N = 5 separate, independent experiments.

The possible effect of CRMP2 on mitochondrial dynamics could be due to its interaction with proteins involved in the regulation of mitochondrial dynamics such as Drp1 and the Miro/Milton complex [27,29,33]. In our experiments, we found that CRMP2 interacted with Drp1 and Miro 2 (Figure 4), proteins involved in mitochondrial fission and motility, respectively [71], but not with FIS1, MFF, syntaphilin, and syntabulin (not shown). The increase in CRMP2 phosphorylation at Thr 509/514 and Ser 522 in HD (Figure 3A,C,D) correlated with a dissociation of CRMP2 from Drp1 and Miro 2 (Figure 4). On the other hand, CRMP2 dephosphorylation at Thr 509/514 and Ser 522 in the presence of (S)-LCM (Figure 3A,C,D) was accompanied by the restored interaction of CRMP2 with Drp1 and Miro 2 (Figure 4). These data suggest that changes in CRMP2 phosphorylation may be linked to alterations in mitochondrial morphology and/or motility.

### 3.3. CRMP2 and Mitochondrial Morphology and Motility in HD

**Figure 5:**
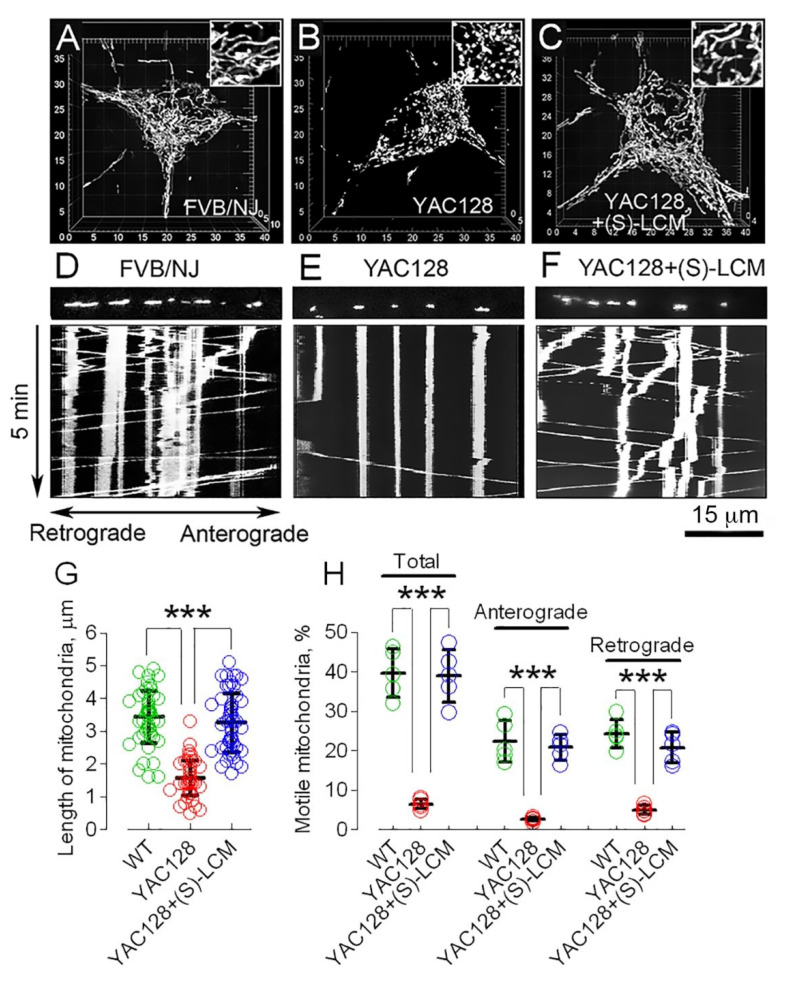
An increase in mitochondrial fission (**A**–**C**,**G**) and suppressed mitochondrial motility (**D**–**F**,**H**) in cultured striatal neurons (10 DIV) from FVB/NJ mice (wild-type, WT) compared to striatal neurons from YAC128 mice. (S)-lacosamide ((S)-LCM) rescued mitochondrial morphology and motility (**C**,**F**–**H**). Mitochondria were visualized by expression of mitochondrially targeted enhanced yellow fluorescent protein (mito-eYFP). (**A**), 3D image shows normal, elongated mitochondria in neuron from wild-type FVB/NJ mouse. (**B**), increased fission in neuron from YAC128 mouse. (**C**), preservation of mitochondrial morphology in YAC128 neuron treated with 10 µM of (S)-LCM for the last 7 days prior to imaging. In *Insets*, mitochondria are shown at additional 2× magnification. In (**D**,**E**), suppressed mitochondrial traffic in cultured striatal neurons from YAC128 mice are compared with neuron from FVB/NJ mouse (WT). (**F**), (S)-LCM rescues mitochondrial motility in neuron from YAC128 mouse. Cells were treated with 10 µM of (S)-LCM for the last 7 days prior to analysis. (**G**), length of mitochondria in μm. One hundred randomly chosen mitochondria from at least 10 neurons from three different platings were analyzed. Data are mean ± SD. (**H**), total percentage of motile mitochondria and mitochondria moving in anterograde and retrograde directions. Data are mean ± SD. *** *p* < 0.001 comparing FVB/NJ neurons (WT) and YAC128 neurons; comparing YAC128 neurons with and without (S)-LCM treatment, N = 5 separate, independent experiments.

Indeed, the changes in CRMP2 phosphorylation at Thr 509/514 and Ser 522 as well as interaction with Drp1 and Miro 2 in HD neurons correlated with alterations in mitochondrial morphology and motility. In experiments with cultured striatal neurons from YAC128 mice and their wild-type littermates (WT), we found the shortening of mitochondria and the suppression of mitochondrial motility in neurons from HD mice (Figure 5A,B,D,E,G,H). The pre-treatment of cultured neurons with (S)-LCM (10 μM for the last 7 days prior to analysis) prevented these changes (Figure 5C,F–H).

### 3.4. CRMP2 and Neuronal Cell Death in HD

**Figure 6:**
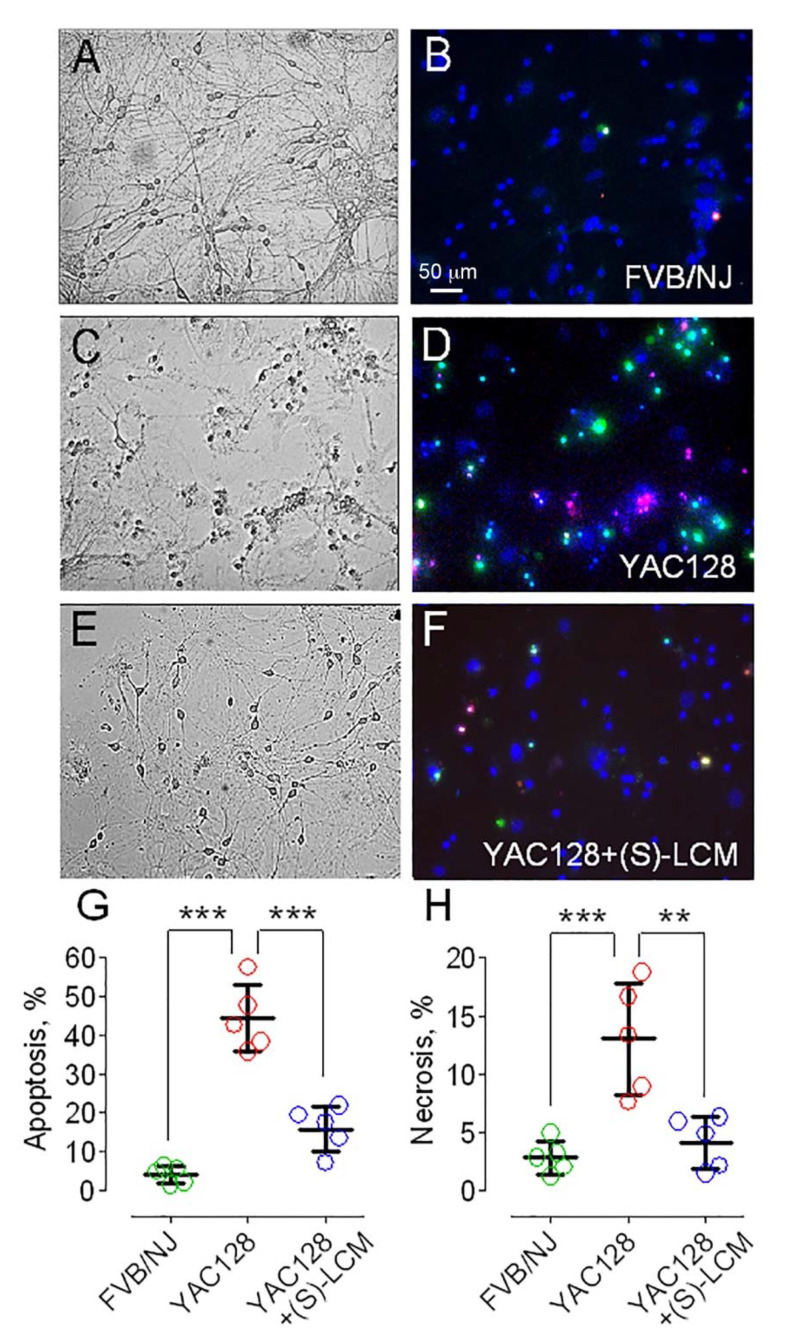
An evaluation of spontaneous necrotic and apoptotic cell death with Chromatin Condensation/Membrane Permeability/Dead Cell Apoptosis Kit (ThermoFisher Scientific, Cat #V23201), containing Hoechst 33342, YO-PRO-1, and propidium iodide (PI). Hoechst 33342 staining (blue fluorescence) indicated live cells. Nuclei staining with PI (red fluorescence) is associated with the loss of barrier properties of the plasma membrane and is considered an indication of necrosis [61]. An induction of apoptosis was evaluated with YO-PRO-1 staining (green staining). These experiments were performed with cultured striatal neurons from FVB/NJ (**B**) and YAC128 (**D**) mice. (S)-lacosamide ((S)-LCM) protected neurons from cell death (**F**). Neurons were cultured for 21 days in vitro (21 DIV). (**A**,**C**), phase contrast bright field images of cultured striatal neurons from FVB/NJ and YAC128 mice, respectively. (**E**), phase contrast bright field image of (S)-LCM-treated cultured striatal neurons from YAC128 mice. (**G**,**H**), quantification of apoptotic cell death and necrotic cell death, respectively. Neurons were treated with 10 µM of (S)-LCM for the last 14 days prior to cell death analysis. Data are mean ± SD. ** *p* < 0.01, *** *p* < 0.001 comparing FVB/NJ neurons and YAC128 neurons; comparing YAC128 neurons with and without (S)-LCM treatment, N = 5 separate, independent experiments.

Alterations in mitochondrial dynamics could be detrimental for neurons. In our experiments, mouse striatal neurons expressing mHTT were more prone to cell death compared with neurons from wild-type mice (Figure 6). Remarkably, pre-treatment with (S)-LCM (10 μM for the last 7 days prior to analysis) noticeably protected neurons expressing mHTT and increased their survival. Thus, the (S)-LCM-induced prevention of CRMP2 hyperphosphorylation at Thr 509/514 and Ser 522 correlated with the restoration of normal mitochondrial morphology and motility and improved the survival of striatal neurons expressing mHTT.

## 4. Discussion

HD is linked to a single mutation of the *Htt* gene and the elongation of poly-glutamine stretch at the N-terminus of HTT protein [1,2]. This apparent simplicity raised the possibility of untangling the pathogenic mechanisms contributing to HD. Mitochondrial damage due to the deleterious action of mHTT is a highly touted etiological factor in HD pathogenesis [3,4,5,6,7,8,9,10]. However, in HD, mitochondria per se may not be damaged by mHTT, and there is a significant body of evidence strongly arguing against the primary etiological factor of mitochondrial bioenergetic deficiency as a contributor to HD pathogenesis [11,12,13,14,15,16,17,18,19]. Moreover, mitochondrial depolarization with the uncoupler 2,4-dinitrophenol was found to be neuroprotective in the N171-82Q HD mouse model [72], contradicting a possible role of mitochondrial dysfunction in HD pathogenesis.

Although mitochondrial bioenergetics could remain intact, alterations in mitochondrial dynamics (mitochondrial morphology and motility) may lead to detrimental consequences for neurons over time [73,74]. In contrast to other cell types, neurons have extended processes connecting neuronal somata to distant synapses. These connections are critical for distant synapses, which require a sufficient energy supply for proper function [73,74]. In addition, these synapses rely on the timely elimination of damaged mitochondria to avoid oxidative injury and defective proteostasis [75]. Both processes require the maintenance of normal mitochondrial morphology and unrestricted mitochondrial motility [76]. Consequently, alterations in mitochondrial dynamics may play a key role in HD pathogenesis.

Mitochondrial morphology and motility are regulated by numerous proteins [27,28,29,30,31,32,33]. Despite significant effort, the exact mechanisms regulating mitochondrial morphology and motility are not completely understood. Some proteins such as Drp1 and Mitofusins are involved in the regulation of mitochondrial morphology [27,28,29]. Other proteins such as kinesins and dyneins are involved in the regulation of mitochondrial motility [32,33]. Interestingly, CRMP2 binds to dynein and kinesin-1 and may regulate their activity [30,31]. In addition, there are proteins involved in both the regulation of mitochondrial morphology and motility, for example, Mitofusin 2 and Miro 2 [77]. Many other proteins are involved in the regulation of mitochondrial morphology and motility [71], but, notwithstanding extensive studies, the entire set of proteins regulating mitochondrial dynamics is not yet completely known. Based on our results, we propose that the phosphoprotein CRMP2 as one of these regulatory proteins.

In our experiments, we found that CRMP2 is hyperphosphorylated in HD. Similar findings were recently reported by Mokhtar et al. [49]. The specificity of CRMP2 pThr 509/514 and pSer 522 was validated in our preliminary unpublished studies (not shown). The antibody for CRMP2 pTyr 32 was validated by Uchida et al. [45]. The antibody against CRMP2 pThr 555 was validated by the commercial supplier (ECM Biosciences (Versailles, KY, USA): https://ecmbio.com/products/CM5391 (accessed on 11 November 2021)).

The different bands in the immunoblots with human postmortem brain tissues shown in Figure 1A might belong to different isoforms of CRMP2, CRMP2A, and CRMP2B, also found by Mokhtar et al. [49], and/or could result from CRMP2 post-translational modifications. In addition, because these immunoblots were produced with postmortem brain tissues, some immunoreactive material might belong to CRMP2 degradation products. Consequently, all shown bands were used for densitometry. The cause of variations in the appearance of bands of different sizes (Figure 1) and why only one band is detectable with human neurons derived from iPSCs (Figure 2) is not clear.

Based on our data, in HD, CRMP2 appears to be hyperphosphorylated at Tyr 32, Thr 509/514, Ser 522, and Thr 555. CRMP2 phosphorylation at Ser 522 by Cdk-5 facilitates subsequent CRMP2 phosphorylation at Thr 509/514 by Gsk-3β [50,51]. Cdk-5 is activated in HD [78]. There are controversial data regarding Gsk-3β expression and activity in HD. Some studies found elevated levels of Gsk-3β in the HD cell model [79], whereas others reported decreased Gsk-3β expression and diminished Gsk-3 signaling in HD [80]. Interestingly, the inhibition of Gsk-3α/β ameliorated HD pathogenesis [81], suggesting that Gsk-3α/β remains active in HD and may lead to detrimental consequences.

Our data indicating the hyperphosphorylation of CRMP2 at Ser 522 and Thr 509/514 in postmortem tissues and in cultured cells expressing mHTT suggest that both Cdk-5 and Gsk-3β are active in HD. Both these kinases, Cdk-5 and Gsk-3β, have multiple targets, and it is possible that the aberrant functioning of these kinases may lead to various detrimental effects. In our study, we found an interesting correlation between CRMP2 phosphorylation in HD and CRMP2 binding to Drp1 and Miro 2, paralleled by alterations in mitochondrial morphology and motility as well as by changes in neuronal survival. Although different pathways might be involved in these processes, the link between CRMP2 phosphorylation and changes in mitochondrial dynamics seems quite possible. It is also possible that alterations in CRMP2 phosphorylation could lead to similar mitochondrial effects in tissues other than brain. However, answering this question requires additional investigation and is beyond the scope of the present study.

In HD, mitochondrial fission is increased [20,21,24,25] and mitochondrial traffic is suppressed [18,20,22,23,26]. The exact mechanisms of these alterations are not completely clear. However, based on our data, it seems conceivable that hyperphosphorylated CRMP2 contributes to these alterations as well as to decreased neuronal survival. The latter is consistent with the well-established correlation between alterations in mitochondrial dynamics and neuronal loss [82,83]. Thus, in the present study, our data identify CRMP2 as a new modulator of mitochondrial dynamics and neuronal survival in HD.

Our current study and previous studies from other investigators [49] found that CRMP2 was hyperphosphorylated in HD, and this appeared to be detrimental for neurons. It was also reported that CRMP2 is hyperphosphorylated in Alzheimer’s disease (AD), and this was correlated with CRMP2 dissociation from kinesin-1 and neuronal cell death [49]. It is possible that the hyperphosphorylation of CRMP2 in HD and AD exerts its detrimental action via multiple mechanisms, including but not limited to changes in interaction with kinesins and dyneins. Our study suggests that CRMP2 hyperphosphorylation in HD and subsequent alterations in interaction with Drp1 and Miro 2 may influence mitochondrial morphology and impede mitochondrial traffic, thus predisposing neurons to cell death. The neuroprotective effect of (S)-LCM, which binds to CRMP2 and prevents its phosphorylation at Thr 509/514 and Ser 522 [50,51], but does not affect CRMP2 phosphorylation at Tyr 32 or Thr 555, or Drp1 phosphorylation at Ser 616, suggests that the changes in CRMP2 phosphorylation at Thr 509/514 and Ser 522 may play a major role in HD neuropathology and, consequently, could be a valid target for therapeutic intervention. Based on the results presented in this paper and on the chemical properties of (S)-LCM, it is conceivable that this agent could potentially be developed into a therapeutic agent.

## Data Availability

Data are contained within the article or supplementary materials.

## Supplementary Materials

The following are available online at https://www.mdpi.com/article/10.3390/cells10113172/s1, Figure S1. The unedited images of immunoblots for Figure 1A. Figure S2. The unedited images of immunoblots for Figure 2G. Figure S3. The unedited images of immunoblots for Figure 3A,B. Figure S4. The unedited images of immunoblots for Figure 4A,B. Table S1. Main characteristics of human postmortem brain samples.
