# Supplementary material for: Involvement of CRMP2 in Regulation of Mitochondrial Morphology and Motility in Huntington’s Disease
Source: Cells. 2021 Nov 15;10(11):3172. doi: 10.3390/cells10113172 (PMC8619197; doi:10.3390/cells10113172)
Supplement: Supplementary file 1 [file cells-10-03172-s001.zip › cells-1419988-supplementary.pdf]

SUPPLEMENTARY MATERIALS

To Figure 1A

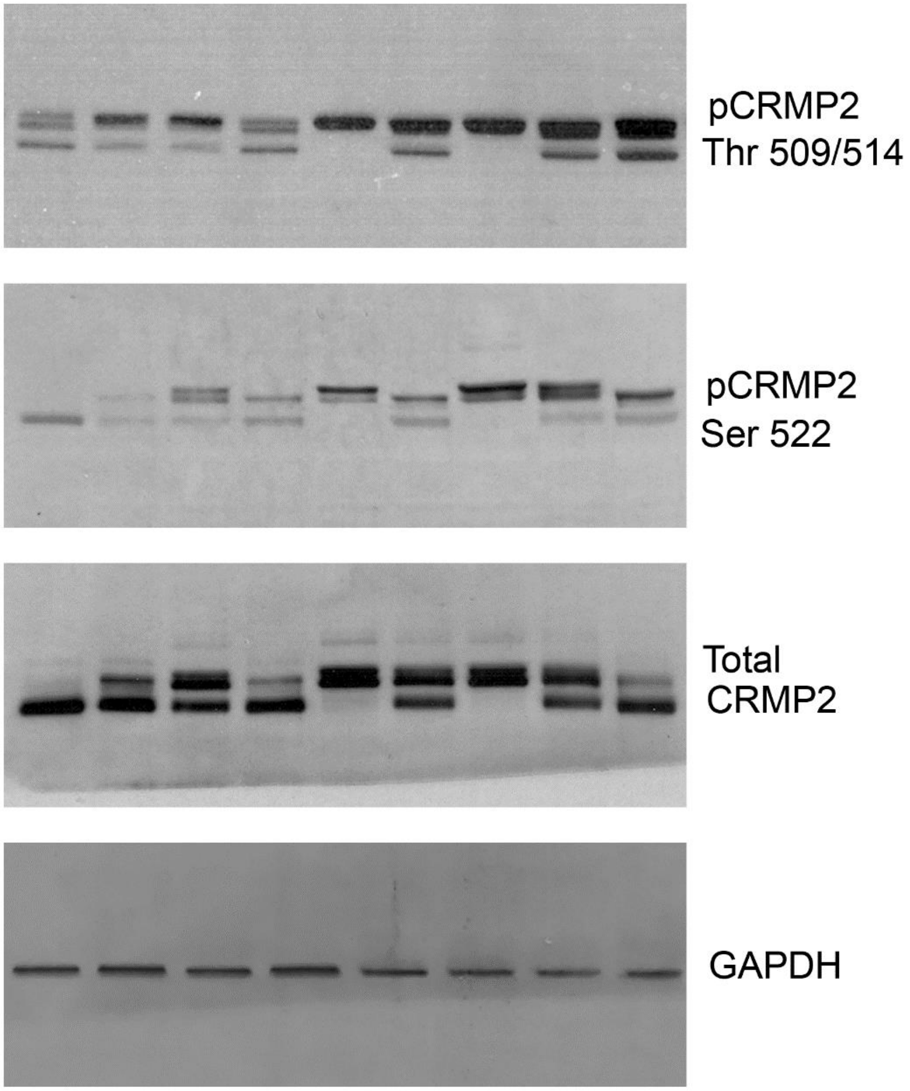

Figure S1. The unedited images of immunoblots for Figure 1A.

To Figure 2G

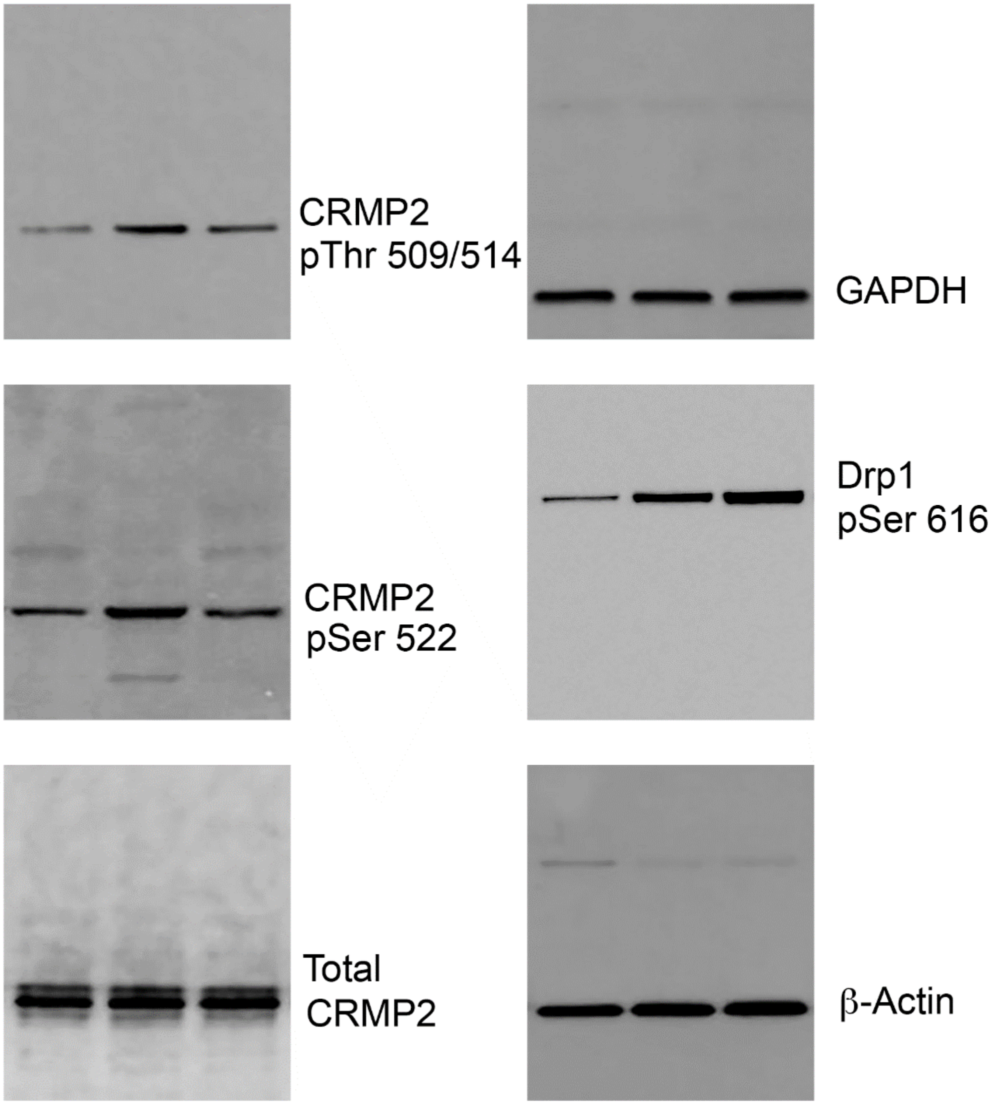

Figure S2. The unedited images of immunoblots for Figure 2G.

To Figure 3A and B

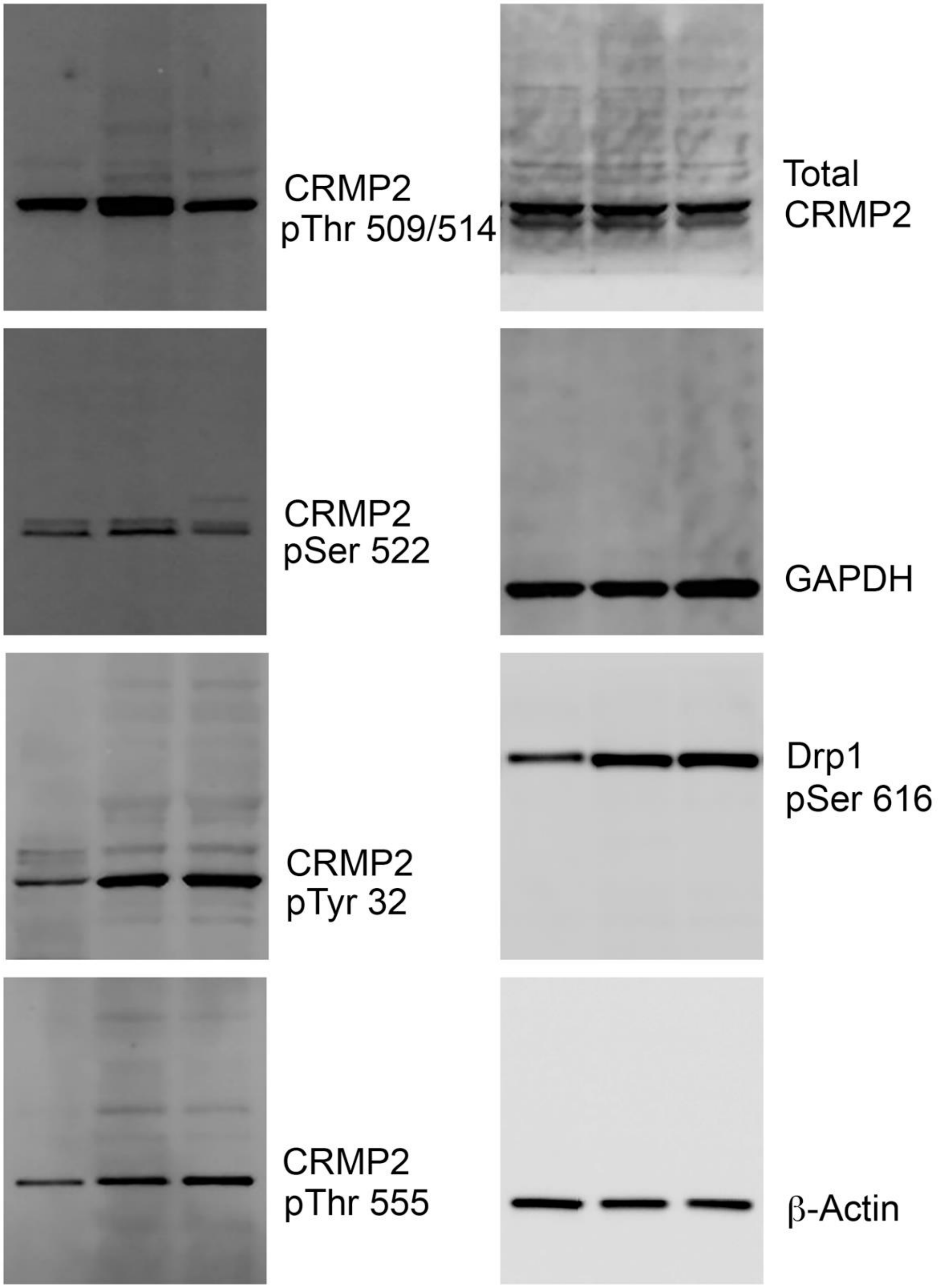

Figure S3. The unedited images of immunoblots for Figure 3A and B.

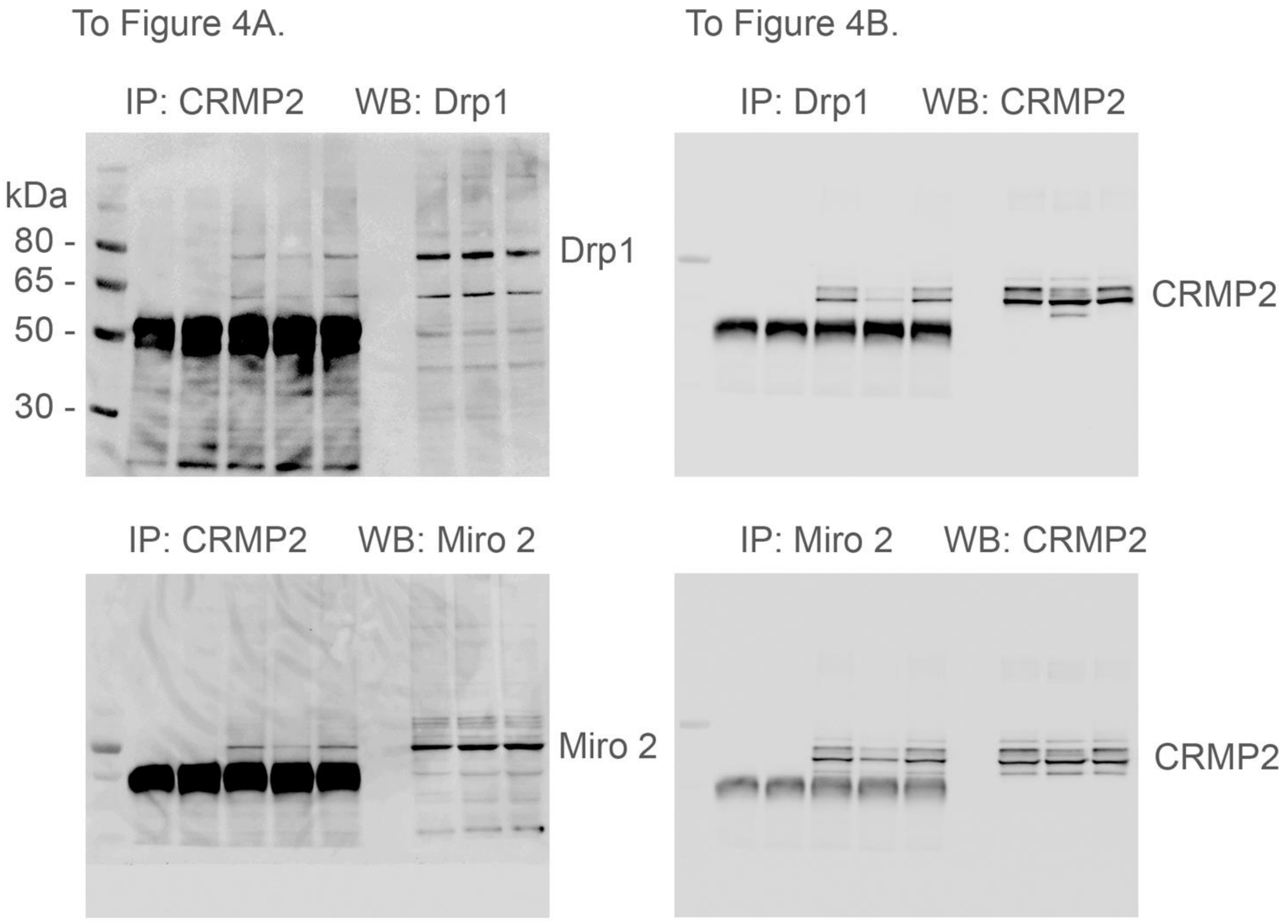

Figure S4. The unedited images of immunoblots for Figure 4A and B.

Supplemental Table S1.

Main characteristics of human postmortem brain samples

Sample ID#	Assigned #	Age	Postmortem Interval (hrs)	Race	Sex	CAG, number	Grade
Control							
5028	1	67	18	W	M	n/d	-
4324	2	62	18	B	F	n/d	-
5404	3	50	17	W	M	n/d	-
5080	4	61	14	B	F	n/d	-
HD Patients							
5199	1	67	13	W	M	44/19	2
5217	2	55	10	W	M	49/21	2
5413	3	43	10	W	M	51/19	2
5104	4	61	16	W	F	47/15	1
4514	5	69	10	W	F	46/17	2-3

n/d – not determined
